# Supplementary material for: A qualitative analysis of female sex workers’ lived experiences with adherence to Pre-exposure Prophylaxis (PrEP) in Zimbabwe
Source: PLoS One. 2025 Sep 4;20(9):e0313366. doi: 10.1371/journal.pone.0313366 (PMC12410797; doi:10.1371/journal.pone.0313366)
Supplement: Attachment — Submitted filename: Reviewer Comments_SK20Jan2025.docx [file pone.0313366.s003.docx]

Review of the manuscript entitled: **A Qualitative Analysis of female sex workers’ lived experiences with adherence to Preexposure Prophylaxis (PrEP) adherence in Zimbabwe.**

Thank you for the opportunity to review the above manuscript. FSWs continue to be at high risk of acquiring HIV. Oral PrEP would certainly help reduce HIV incidence in this population if adherence challenges could be dealt with.

Below are some comments:

**Title:** Would modifying to include challenges and strategies to improve adherence be better?

The word adherence is repeated.

**Abstract:**

- Confirm if 10% is prevalence
- What form of PrEP is being referred to? Oral, Injectable or Ring?

**Introduction:**

- Redefine to Oral PrEP since there are currently various forms of PrEP.
- Also indicate if Truvada (TDF/FTC) since Descovy is available, but approved for MSM
- In the systematic review, what were some of the reasons for discontinuation?
- What are the negative consequences being referred to in the study done in Zimbabwe?
- What would difficulty in understanding PrEP refer to? Understanding the prescription?
- Please clarify the research gap this paper is trying to address as this is not clear from the background provided.

**Methods:**

- Study design: Was this Cross-sectional or longitudinal? Were any repeat interviews done?
- What was the age group required for the study? What demographics were used for participant selection?
- Were there any refusals during recruitment? What were the reasons?
- Were any notes taken during the interviews?
- Was coding done manually? By how many people? 20 scripts are quite many for one person. Was software used?
- What was the working definition of PrEP adherence?
- Ethical considerations: Were all the FSWs literate?

**Results:**

- Table 1: Please provide a more comprehensive title for the table
- The characteristic/item for marital status is not provided.
- The quotations provided seem many per sub-theme. Were there any differences per age group or other demographic? Some of the quotes bring out the same points. E.g under forgetfulness- a pill buddy appears twice in the same age group; under side effects, change of time also appears twice in the same age group; most GBV is in the same age group and similar.
- Probably two per theme would suffice

**Discussion/conclusion:**

- Review the spelling of urgency instead of agency; strife instead of strive?
- What strategies were use d in the SWOP study?
- Please confirm that these differentiated service delivery of PrEP is not part of the Zimbabwe guidelines. Many countries adopted these after WHO passed them.
- Under recommendations, what support is being suggested? Please clarify
