# Supplementary material for: A qualitative analysis of female sex workers’ lived experiences with adherence to Pre-exposure Prophylaxis (PrEP) in Zimbabwe
Source: PLoS One. 2025 Sep 4;20(9):e0313366. doi: 10.1371/journal.pone.0313366 (PMC12410797; doi:10.1371/journal.pone.0313366)
Supplement: Attachment — Submitted filename: Response to Reviewers.docx [file pone.0313366.s005.docx]

**Plos One response to reviewer**

**Manuscript title: A Qualitative Analysis of female sex workers’ lived experiences with adherence to Preexposure Prophylaxis (PrEP) adherence in Zimbabwe.**

| **Comment** | **Response** | **Pagination** |
| --- | --- | --- |
| **Title:** |  |  |
| **Reviewer 1** |  |  |
| Would modifying to include challenges and strategies to improve adherence be better?  The word adherence is repeated. | We have removed the repetition on adherence to read “A Qualitative Analysis of female sex workers’ lived experiences with adherence to Preexposure Prophylaxis (PrEP) in Zimbabwe”. | P:1  Line 2 |
| **Abstract:** |  |  |
| Confirm if 10% is prevalence | We confirm that 10% refers to HIV incidence. | Page:1  Line 20 |
| What form of PrEP is being referred to? Oral, Injectable or Ring? | We are referring to oral PrEP. This was previously defined from the introduction: “...an HIV prevention option taken as a daily pill”. | Page:1  Line 21 |
| **Introduction:** |  |  |
| Redefine to Oral PrEP since there are currently various forms of PrEP. | We defined oral PrEP in the introduction section. The second paragraph starts with defining oral PrEP. | Page: 3  Paragraph 2  Line 63 -64 |
| Also indicate if Truvada (TDF/FTC) since Descovy is available, but approved for MSM | We have clarified that this is Truvada - TDF/FTC.. We have added the following statement: “The oral PrEP containing Tenofovir - TDF is recommended for use. In Zimbabwe TDF/FTC is preferred, with TDF/3TC as an alternative” | Page: 3  Paragraph: 2  Line 66-67 |
| In the systematic review, what were some of the reasons for discontinuation? | We have summarized some of the reported reasons for discontinuation as follows:  “The reasons for PrEP discontinuation were reported as side effects, stigma, influence of partners, difficulty in accessing services, and reduced HIV-risk perception. [ | Page: 3  Paragraph: 3  Lines 77-78 |
| What are the negative consequences being referred to in the study done in Zimbabwe? | We have clarified the negative consequences as follows: “Female sex workers were faced with negative family discouragement, skepticism, and fear of being given experimental drugs with no proven efficacy”. | Page:4  Paragraph: 4  Lines: 85-88 |
| What would difficulty in understanding PrEP refer to? Understanding the prescription? | We have clarified that difficulty in understanding PrEP referred to a lack of complete understanding that oral PrEP needs to be taken during periods of risk. We have replaced the text as follows;  “Female sex workers reported lack of understanding regarding taking and adhering to PrEP as prescribed”. | Page:3  Paragraph: 4  Lines: 87-88 |
| Please clarify the research gap this paper is trying to address as this is not clear from the background provided. | The research gap is highlighted in the following text; “This article aims to highlight the FSWs’ psychosocial related experiences on PrEP adherence that could be used to inform the development of a psychosocial intervention – which was the main aim of a bigger study”..  We deleted the following text “this study sought to understand the current lived realities of FSWs with oral PrEP adherence with a view to identifying the key elements to enhance continuation. on page: lines: 113-114 | Page: 4  Paragraph: 2  Lines: 113-116 |
| **Methods:** |  |  |
| Study design: Was this Cross-sectional or longitudinal? Were any repeat interviews done? | This was a cross sectional study with no repeat interviews conducted to understand the lived experiences of PrEP adherence among FSWs on oral PrEP. The following text was added, “These qualitative interviews were conducted once-off, with no repeat interviews held” | Page: 5  Paragraph: 2  Lines: 130-131 |
| What was the age group required for the study? What demographics were used for participant selection? | We have added the age and eligibility criteria for qualitative interviews as follows. “To be eligible, one had to be 18 years or older, identify as a female sex worker, be on oral PrEP” | Page: 5  Paragraph: 3  Lines: 136-137 |
| Were there any refusals during recruitment? What were the reasons? | We have added the following text,  “The Female sex workers presenting at the clinic for their first PrEP follow-up visit were informed about the study, and invited the FSWs to participate in the study. The potential participants were given the opportunity to refuse to participate. However, once the study was explained thoroughly, all participants who met the criteria were recruited until data saturation was reached.” | Page: 6  Paragraph: 1  Lines: 148-153 |
| Were any notes taken during the interviews? | Detailed notes were taken during the study, and the interviews were all audio recorded. This is explained as follows: “Detailed notes were taken during the interview. In addition to the notes, all interviews were audio recorded to ensure accuracy in data capturing”. | Page: 7  Paragraph: 1  Lines: 1776-177 |
| Was coding done manually? By how many people? 20 scripts are quite many for one person. Was software used? | The researcher coded the interviews manually with a peer, for intercoder agreement. In addition to this, there was oversight provided by the supervisor who is a qualitative expert. This is explained as follows: “All the coding was done manually by two researchers, the researcher and a fellow qualitative researcher, using an excel sheet. The supervisor, who is a qualitative expert, provided oversight of the coding and analysis process”. | Page: 8  Paragraph: 1  Line: 219-221 |
| What was the working definition of PrEP adherence? | This text has been added: “PrEP adherence was defined as the taking of PrEP during periods of risk. As these were FSWs, the assumption was that their risk was continuous”. | Pages: 7  Paragraph: 1  Lines 180-181 |
| Ethical considerations: Were all the FSWs literate? | We have added the following text, “The FSWs participating in the study were literate, and they had an option to use the English language or their venarcular. The literacy rates in Zimbabwe are higher than other African countries ”. | Pages: 9  Paragraph: 2  Lines: 248-251 |
| **Results:** |  |  |
| Table 1: Please provide a more comprehensive title for the table | The following text has been added “Demographic Characteristics of FSWs on PrEP who participated in the study” | Pages: 9  Paragraph: 4  Lines: 262-263 |
| The characteristic/item for marital status is not provided. | We have added the item for “marital status” | Pages: 10  Paragraph: 4  Lines: 263 |
| The quotations provided seem many per sub-theme. Were there any differences per age group or other demographic? Some of the quotes bring out the same points. E.g under forgetfulness- a pill buddy appears twice in the same age group; under side effects, change of time also appears twice in the same age group; most GBV is in the same age group and similar. | We note the observation. However, the intention was not to compare themes or experiences per age group as done by the reviewer, but to analyse and identify data that has similar meaning for the development of appropriate themes. |  |
| Probably two per theme would suffice | We have reduced the quotes to two per theme as per the reviewer’s advice. |  |
| **Discussion/conclusion:** |  |  |
| Review the spelling of urgency instead of agency; strife instead of strive? | We have reviewed the spellings of “agency’, which meant, the FSWs had a sense of “control”. We have left it as it was.  We have also reviewed the spelling of “strive”, and replaced it with “strife”, as this was referring to periods of difficulty, the FSWs were experiencing during COVID 19. | Pages: 17  Paragraph: 2  Lines: 469 |
| What strategies were used in the SWOP study? | The SWOP study applied strategies around the establishment and implementation of effective outreach and peer education plans. We have added the following text, “The SWOP study used two main strategies, namely, community outreach and peer education”. | Pages: 17  Paragraph: 1  Lines: 461- 462 |
| Please confirm that these differentiated service delivery of PrEP is not part of the Zimbabwe guidelines. Many countries adopted these after WHO passed them. | These differentiated service delivery of PrEP are part of the Zimbabwe guidelinese adapted from the UNAIDS guidelines We have added the following text; “Zimbabwe went on to adapt the UNAIDS guidance through it’s Operational Services Delivery Manual for the Prevention, Care and Treatment of HIV in Zimbabwe (2022)”. | Pages: 17  Paragraph: 2  Lines: 475-477 |
| Under recommendations, what support is being suggested? Please clarify | We have added the following text to clarify the support we are suggesting. “Additionally, we recommend outreach services for FSWs as a way of bringing the PrEP services to where the FSWs are, and the use of peer support to encourage persistent PrEP use”. | Pages: 18  Paragraph: 2  Lines: 497-499 |
|  |  |  |
| **Reviewer 2** |  |  |
| **Introduction:** |  |  |
| Please state the theoretical orientation that informed your results and describe it applicability. | The findings were informed by a feminist lens. | Page: 8  Paragraph: 1 |
| **Results:** |  | Lines: 225 |
| In the negative theme, you talk about GBV and Stigma. I am wondering whether these are two separate experiences or the same. Please revise and clarify what you are trying to put forward. | We note the observation. However, in our analysis, the stigma was associated with taking PrEP. This stigma could be from partners or any other community members. GBV, in particular was related to violence against FSWs once discovered to be using “ARVs” thus exposing the partner to the virus. |  |
| The narratives in this section are insufficient to guide the reader in understanding how the findings affected the participants. | The direct quotations help bring to life the FSW experiences from their lenses and not from the researcher’s lens. We hope this helps give some vivid descriptions of the untold stories and experiences of FSWs on oral PrEP. |  |
| **Discussion:** |  |  |
| What were the strengths and weaknesses of your study? | We have added a new section on the study strengths and weaknesses. | Pages: 18  Paragraph: 2  Lines: 501-509 |
